# A New Family of Giardial Cysteine-Rich Non-VSP Protein Genes and a Novel Cyst Protein

**DOI:** 10.1371/journal.pone.0000044

**Published:** 2006-12-20

**Authors:** Barbara J. Davids, David S. Reiner, Shanda R. Birkeland, Sarah P. Preheim, Michael J. Cipriano, Andrew G. McArthur, Frances D. Gillin

**Affiliations:** 1 Department of Pathology, Division of Infectious Diseases, University of California San Diego, California, United States of America; 2 Josephine Bay Paul Center for Comparative Molecular Biology and Evolution, Marine Biological Laboratory Woods Hole, Massachusetts, United States of America; 3 Department of Biology, Woods Hole Oceanographic Institution Woods Hole, Massachusetts, United States of America; Utrecht University, Netherlands

## Abstract

Since the *Giardia lamblia* cyst wall is necessary for survival in the environment and host infection, we tested the hypothesis that it contains proteins other than the three known cyst wall proteins. Serial analysis of gene expression during growth and encystation revealed a gene, “HCNCp” (High Cysteine Non-variant Cyst protein), that was upregulated late in encystation, and that resembled the classic *Giardia* variable surface proteins (VSPs) that cover the trophozoite plasmalemma. HCNCp is 13.9% cysteine, with many “CxxC” tetrapeptide motifs and a transmembrane sequence near the C-terminus. However, HCNCp has multiple “CxC” motifs rarely found in VSPs, and does not localize to the trophozoite plasmalemma. Moreover, the HCNCp C-terminus differed from the canonical VSP signature. Full-length epitope-tagged HCNCp expressed under its own promoter was upregulated during encystation with highest expression in cysts, including 42 and 21 kDa C-terminal fragments. Tagged HCNCp targeted to the nuclear envelope in trophozoites, and co-localized with cyst proteins to encystation-specific secretory vesicles during encystation. HCNCp defined a novel trafficking pathway as it localized to the wall and body of cysts, while the cyst proteins were exclusively in the wall. Unlike VSPs, HCNCp is expressed in at least five giardial strains and four WB subclones expressing different VSPs. Bioinformatics identified 60 additional large high cysteine membrane proteins (HCMp) containing ≥20 CxxC/CxC's lacking the VSP-specific C-terminal CRGKA. HCMp were absent or rare in other model or parasite genomes, except for *Tetrahymena thermophila* with 30. MEME analysis classified the 61 gHCMp genes into nine groups with similar internal motifs. Our data suggest that HCNCp is a novel invariant cyst protein belonging to a new HCMp family that is abundant in the *Giardia* genome. HCNCp and the other HCMp provide a rich source for developing parasite-specific diagnostic reagents, vaccine candidates, and subjects for further research into *Giardia* biology.

## Introduction

Giardiasis is a major contributor to the enormous burden of human diarrheal diseases, which are second only to respiratory infections as causes of mortality and morbidity worldwide [1]–[3]. Although diarrheal diseases rarely kill children in the USA, they remain important causes of infant death in less-developed countries [2], [4], [5]. Unlike the rotaviruses, which affect infants, giardiasis can also hamper adults in their productive years. In the USA, *G. lamblia* has been one of the most frequently identified causes of water-borne intestinal disease, and causes diarrhea in daycare centers and among hikers and campers. Nonetheless, the basic biology and pathogenesis of this parasite remain incompletely understood. Although trophozoites do not invade or secrete any known toxin [2], [6], they can cause severe diarrhea that can cause failure of children to thrive. However, about half of infected people lack symptoms and the infection often resolves without treatment. Thus, both the duration and symptoms of giardiasis are highly variable in immunocompetent people for reasons that are not understood.

No giardial virulence factor is known and this protist's ability to survive in diverse, hostile environments may be a key to its pathophysiology. To survive within the small intestine, *Giardia* trophozoites are covered with a single layer of an unusual Type 1 integral membrane protein that protects them from proteolysis [7]. Normally, each trophozoite expresses only one such surface protein from approximately 150 in the genome and switching of these variant-specific surface proteins (VSPs) [8]–[10] likely protects the trophozoite surface from both immunologic and environmental attack. VSP gene expression can spontaneously switch both *in vitro* and *in vivo* [11], [12] every six to thirteen generations [13]. Most isolates are mixed populations of trophozoites expressing different VSPs. Immune [11] or environmental [14] stress *in vitro* can select for or against expression of different VSPs, which are usually not immunologically cross-reactive [15], suggesting that a major function of antigenic variation is immune evasion. VSPs are extremely (∼12%) cysteine-rich, with most Cys residues in a CxxC motif, where x can be any amino acid [16], [17]. Although VSPs differ in size and sequence, they are characterized by a highly conserved C-terminal membrane spanning region, a hydrophilic cytoplasmic tail with a conserved five amino acid CRGKA signature sequence, and an extended polyadenylation signal [7], [8], [18].

If trophozoites are carried downstream in the intestine, they must encyst in order to survive outside the host [8]. Encystation is characterized by construction of an extracellular cyst wall which protects the parasite from hypotonic lysis in the external environment and from gastric acid when infecting a new host [8], [19], [20]. The *Giardia*' cyst wall is biologically important as a simple model extracellular matrix, with both structural and signal transduction functions [21], [22]. Encystation contributes to immune evasion [23] by two distinct pathways: first, the cyst wall covers the trophozoite surface during encystation and sequesters it from host defenses and second, encystation leads to switching of VSPs [18], [24], [25]. Despite its importance, the composition, architecture, and formation of the cyst wall are poorly understood. Only three structural cyst wall proteins (CWPs) have been identified [26]–[28]. CWPs 1–3, which do not undergo antigenic variation, have 14–16 Cys residues and similar leucine rich repeats (LRRs). In addition to CWPs, the cyst wall is also composed of fibrils of poly N-acetylgalactosamine [29]–[31].

The VSPs and CWPs define distinct expression and trafficking patterns [26]–[28], [32]. A well-studied VSP called TSA 417 was highly expressed during vegetative growth and transported by a conventional ER pathway to the plasmalemma [32]. TSA 417 was downregulated and the protein internalized during encystation. In contrast, CWPs are only expressed during encystation and are exclusively transported to the cyst wall within the matrix of novel regulated encystation secretory vesicles (ESVs) [33]–[36]. They do not localize to the plasma membrane. Neither the ESVs nor the cyst wall contains VSPs.

In theses studies, we tested the hypothesis that the cyst wall may contain additional proteins. Recently, our ongoing transcriptome analyses of the giardial life cycle revealed a gene encoding an unusual VSP-like protein that was upregulated during encystation. Functional and bioinformatic analyses showed that this gene encodes a divergent cysteine-rich protein (HCNCp) that is increasingly expressed in the cyst cell body and cyst wall during encystation and does not vary among giardial isolates. HCNCp may provide a functional link between immune avoidance and differentiation.

## Results

### Identification of HCNCp

**Figure 1:**
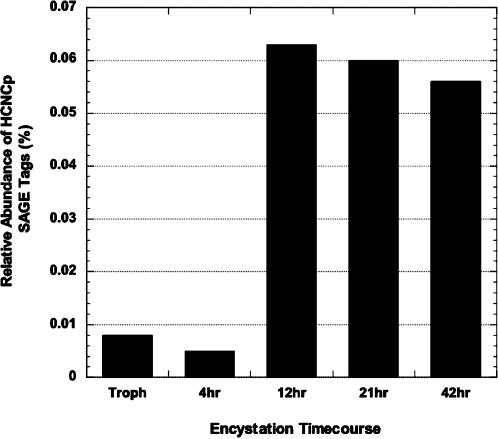
HCNCp mRNA Transcripts Are Upregulated During Encystation SAGE revealed increased steady state abundance of HCNCp transcripts beginning at 12 hr encystation through 42 hr compared to trophozoite (“troph”) and 4 hr encystation. SAGE data is presented as percentage of all tags sampled at a given time point.

A major goal of the giardial transcriptome analysis was to identify new genes whose expression patterns suggest an important role in differentiation. Correcting for sequencing error, we sampled 36,000–39,000 SAGE tags throughout the *Giardia lamblia* life cycle, except for 21 hours encystation, in which we were only able to sample 18,370 tags. 83.6% of all 15 bp SAGE tag sequences could be mapped to a single location in the 12 Mbp “giardia14” genome assembly, with an additional 9.0% mapping to multiple locations primarily due to duplicated genes. In addition to numerous upregulated transcripts, we identified ORF 40376 (originally annotated as a large VSP) as a gene significantly upregulated from 12 to 42 hr of encystation (Figure 1). Northern analyses confirmed an increase in the transcript for this gene during encystation (data not shown). ORF 40376 encodes a large gene of 3790 bp (1609 amino acids) and the encoded protein has a predicted molecular weight of 169,301.7 kDa. We named the gene product High Cysteine Non-variant Cyst protein (HCNCp) (GenBank accession DQ144994). Like the VSPs, HCNCp is a strikingly cysteine-rich (13.9%), acidic (theoretical pI 4.72), Type 1 integral membrane protein with multiple CxxC and GGCY motifs (Tables 1 and 3), although it did not have a Zn finger motif. It has a typical N-terminal signal peptide sequence whose predicted cleavage site is between amino acids 14 and 15. Also like the VSPs, HCNCp has a single C-terminal membrane spanning region and short cytoplasmic tail. However, the predicted C-terminal transmembrane (TM) region of HCNCp is divergent from those of the VSPs (1577-GAITGIAIAVIAVIGCAVGVLVW-1599) with a similar, but distinct amino acid tail, CRRSKAV for HCNCp versus CRGKA for the VSPs (Table 1). While VSPs utilize a unique extended polyadenylation signal [8], [18], the polyadenylation signal for HCNCp matches that of the majority of non-VSP genes within the *G. lamblia* genome (AGTAAAT). MEME analysis did not find a unique putative promoter sequence shared between HCNCp and the VSPs or CWPs, although HCNCp did share a typical AT-rich putative transcription initiation signal with the CWPs. There was no evidence of the C(T/A)ACAG promoter sequence associated with gMyb2 [37], a Myb-related protein involved in upregulation of CWPs and G6PI-B [38] during encystation.

**Table 1:**
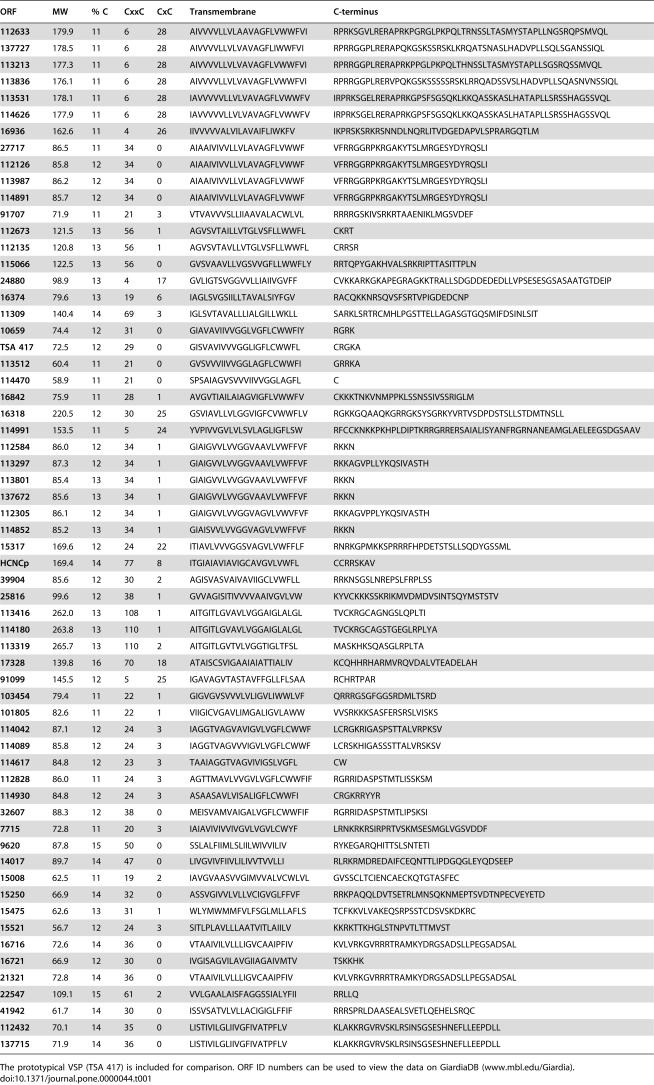
HCMp Proteins In The Giardial Genome Ordered By Transmembrane Region

ORF	MW	% C	CxxC	CxC	Transmembrane	C-terminus
**112633**	179.9	11	6	28	AIVVVVLLVLAAVAGFLVWWFVI	RPRKSGVLRERAPRKPGRGLPKPQLTRNSSLTASMYSTAPLLNGSRQPSMVQL
**137727**	178.5	11	6	28	AIVVVVLLVLVAVAGFLIWWFVI	RPRRGGPLRERAPQKGSKSSRSKLKRQATSNASLHADVPLLSQLSGANSSIQL
**113213**	177.3	11	6	28	AIVVVVLLVLVAVAGFLVWWFVI	RPRRGGPLRERAPRKPPGLPKPQLTHNSSLTASMYSTAPLLSGSRQSSMVQL
**113836**	176.1	11	6	28	AIVVVVLLVLVAVAGFLVWWFVI	RPRRGGPLRERVPQKGSKSSSSSRSKLRRQADSSVSLHADVPLLSQASNVNSSIQL
**113531**	178.1	11	6	28	IAVVVVVLLVLVAVAGFLVWWFV	IRPRKSGELRERAPRKGPSFSGSQKLKKQASSKASLHATAPLLSRSSHAGSSVQL
**114626**	177.9	11	6	28	IAVVVVVLLVLVAVAGFLVWWFV	IRPRKSGELRERAPRKGPSFSGSQKLKKQASSKASLHATAPLLSRSSHAGSSVQL
**16936**	162.6	11	4	26	IIVVVVVALVILAVAIFLIWKFV	IKPRSKSRKRSNNDLNQRLITVDGEDAPVLSPRARGQTLM
**27717**	86.5	11	34	0	AIAAIVIVVLLVLAVAGFLVWWF	VFRRGGRPKRGAKYTSLMRGESYDYRQSLI
**112126**	85.8	12	34	0	AIAAIVIVVLLVLAVAGFLVWWF	VFRRGGRPKRGAKYTSLMRGESYDYRQSLI
**113987**	86.2	12	34	0	AIAAIVIVVLLVLAVAGFLVWWF	VFRRGGRPKRGAKYTSLMRGESYDYRQSLI
**114891**	85.7	12	34	0	AIAAIVIVVLLVLAVAGFLVWWF	VFRRGGRPKRGAKYTSLMRGESYDYRQSLI
**91707**	71.9	11	21	3	VTVAVVVSLLIIAAVALACWLVL	RRRRGSKIVSRKRTAAENIKLMGSVDEF
**112673**	121.5	13	56	1	AGVSVTAILLVTGLVSFLLWWFL	CKRT
**112135**	120.8	13	56	1	AGVSVTAVLLVTGLVSFLLWWFL	CRRSR
**115066**	122.5	13	56	0	GVSVAAVLLVGSVVGFLLWWFLY	RRTQPYGAKHVALSRKRIPTTASITTPLN
**24880**	98.9	13	4	17	GVLIGTSVGGVVLLIAIIVGVFF	CVKKARKGKAPEGRAGKKTRALLSDGDDEDEDLLVPSESESGSASAATGTDEIP
**16374**	79.6	13	19	6	IAGLSVGSIILLTAVALSIYFGV	RACQKKNRSQVSFSRTVPIGDEDCNP
**11309**	140.4	14	69	3	IGLSVTAVALLLIALGILLWKLL	SARKLSRTRCMHLPGSTTELLAGASGTGQSMIFDSINLSIT
**10659**	74.4	12	31	0	GIAVAVIIVVGGLVGFLCWWFIY	RGRK
**TSA 417**	72.5	12	29	0	GISVAVIVVVGGLIGFLCWWFL	CRGKA
**113512**	60.4	11	21	0	GVSVVVIIVVGGLAGFLCWWFI	GRRKA
**114470**	58.9	11	21	0	SPSAIAGVSVVVIIVVGGLAGFL	C
**16842**	75.9	11	28	1	AVGVTIAILAIAGVIGFLVWWFV	CKKKTNKVNMPPKLSSNSSIVSSRIGLM
**16318**	220.5	12	30	25	GSVIAVLLVLGGVIGFCVWWFLV	RGKKGQAAQKGRRGKSYSGRKYVRTVSDPDSTSLLSTDMTNSLL
**114991**	153.5	11	5	24	YVPIVVGVLVLSVLAGLIGFLSW	RFCCKNKKPKHPLDIPTKRRGRRERSAIALISYANFRGRNANEAMGLAELEEGSDGSAAV
**112584**	86.0	12	34	1	GIAIGVVLVVGGVAAVLVWFFVF	RKKN
**113297**	87.3	12	34	1	GIAIGVVLVVGGVAAVLVWFFVF	RKKAGVPLLYKQSIVASTH
**113801**	85.4	13	34	1	GIAIGVVLVVGGVAAVLVWFFVF	RKKN
**137672**	85.6	13	34	1	GIAIGVVLVVGGVAAVLVWFFVF	RKKN
**112305**	86.1	12	34	1	GIAIGVVLVVGGVAGVLVWVFVF	RKKAGVPPLYKQSIVASTH
**114852**	85.2	13	34	1	GIAISVVLVVGGVAGVLVWFFVF	RKKN
**15317**	169.6	12	24	22	ITIAVLVVVGGSVAGVLVWFFLF	RNRKGPMKKSPRRRFHPDETSTSLLSQDYGSSML
**HCNCp**	169.4	14	77	8	ITGIAIAVIAVIGCAVGVLVWFL	CCRRSKAV
**39904**	85.6	12	30	2	AGISVASVAIVAVIIGCLVWFLL	RRKNSGSLNREPSLFRPLSS
**25816**	99.6	12	38	1	GVVAGISITIVVVVAAIVGVLVW	KYVCKKKSSKRIKMVDMDVSINTSQYMSTSTV
**113416**	262.0	13	108	1	AITGITLGVAVLVGGAIGLALGL	TVCKRGCAGNGSLQPLTI
**114180**	263.8	13	110	1	AITGITLGVAVLVGGAIGLALGL	TVCKRGCAGSTGEGLRPLYA
**113319**	265.7	13	110	2	AITGITLGVTVLVGGTIGLTFSL	MASKHKSQASGLRPLTA
**17328**	139.8	16	70	18	ATAISCSVIGAAIAIATTIALIV	KCQHHRHARMVRQVDALVTEADELAH
**91099**	145.5	12	5	25	IGAVAGVTASTAVFFGLLFLSAA	RCHRTPAR
**103454**	79.4	11	22	1	GIGVGVSVVVLVLIGVLIWWLVF	QRRRGSGFGGSRDMLTSRD
**101805**	82.6	11	22	1	VIIGICVGAVLIMGALIGVLAWW	VVSRKKKSASFERSRSLVISKS
**114042**	87.1	12	24	3	IAGGTVAGVAVIGVLVGFLCWWF	LCRGKRIGASPSTTALVRPKSV
**114089**	85.8	12	24	3	IAGGTVAGVVVIGVLVGFLCWWF	LCRSKHIGASSSTTALVRSKSV
**114617**	84.8	12	23	3	TAAIAGGTVAGVIVIGSLVGFL	CW
**112828**	86.0	11	24	3	AGTTMAVLVVGVLVGFLCWWFIF	RGRRIDASPSTMTLISSKSM
**114930**	84.8	12	24	3	ASAASAVLVISALIGFLCWWFI	CRGKRRYYR
**32607**	88.3	12	38	0	MEISVAMVAIGALVGFLCWWFIF	RGRRIDASPSTMTLIPSKSI
**7715**	72.8	11	20	3	IAIAVIVIVVIVGVLVGVLCWYF	LRNKRKRSIRPRTVSKMSESMGLVGSVDDF
**9620**	87.8	15	50	0	SSLALFIIMLSLIILWIVVILIV	RYKEGARQHITTSLSNTETI
**14017**	89.7	14	47	0	LIVGVIVFIIVLILIVVTVVLLI	RLRKRMDREDAIFCEQNTTLIPDGQGLEYQDSEEP
**15008**	62.5	11	19	2	IAVGVAASVVGIMVVALVCWLVL	GVSSCLTCIENCAECKQTGTASFEC
**15250**	66.9	14	32	0	ASSVGIVVLVLLVCIGVGLFFVF	RRKPAQQLDVTSETRLMNSQKNMEPTSVDTNPECVEYETD
**15475**	62.6	13	31	1	WLYMWMMFVLFSGLMLLAFLS	TCFKKVLVAKEQSRPSSTCDSVSKDKRC
**15521**	56.7	12	24	3	SITLPLAVLLLAATVITLAIILV	KKRKTTKHGLSTNPVTLTTMVST
**16716**	72.6	14	36	0	VTAAIVILVLLLIGVCAAIPFIV	KVLVRKGVRRRTRAMKYDRGSADSLLPEGSADSAL
**16721**	66.9	12	30	0	IVGISAGVILAVGIIAGAIVMTV	TSKKHK
**21321**	72.8	14	36	0	VTAAIVILVLLLIGVCAAIPFIV	KVLVRKGVRRRTRAMKYDRGSADSLLPEGSADSAL
**22547**	109.1	15	61	2	VVLGAALAISFAGGSSIALYFII	RRLLQ
**41942**	61.7	14	30	0	ISSVSATVLVLLACIGIGLFFIF	RRRSPRLDAASEALSVETLQEHELSRQC
**112432**	70.1	14	35	0	LISTIVILGLIIVGFIVATPFLV	KLAKKRGVRVSKLRSINSGSESHNEFLLEEPDLL
**137715**	71.9	14	36	0	LISTIVILGLIIVGFIVATPFLV	KLAKKRGVRVSKLRSINSGSESHNEFLLEEPDLL

The prototypical VSP (TSA 417) is included for comparison. ORF ID numbers can be used to view the data on GiardiaDB (www.mbl.edu/Giardia).

### HCNCp Protein Is Upregulated During Encystation and Does Not Traffic to the Plasma Membrane

**Figure 2:**
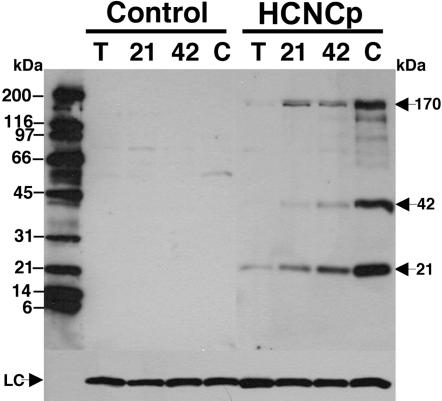
Expression of HCNCp During Differentiation Full length epitope-tagged HCNCp protein (arrow at 170 kDa) was detected beginning at 21 hr encystation (“21”) and was at peak levels at the cyst (“C”) stage. Proteins having an AU1 tag and relative molecular masses of 42 and 21 kDa also increased during encystation (arrows). Anti-AU1 antibodies did not react to non-transfected “control” trophozoites (“T”) or encysting cells (“21”, “42”, or “C”). Size markers are indicated by dashes on the left side of figure in kDa and the taglin loading control is shown at the bottom of the figure (“LC”).

Based on its degree of similarity to the VSPs, we tested the hypothesis that HCNCp is a large variable cysteine-rich protein increasingly expressed on the trophozoite surface during encystation. We expressed full-length (∼170 kDa) HCNCp under its own promoter with a small C-terminal epitope-tag to evaluate protein expression profiles. Full-length HCNCp was faintly detected in trophozoites, but increasingly expressed at 21 and 42 hr of encystation and highest in water-resistant cysts (Figure 2). The protein expression profile of HCNCp is similar to its stable mRNA levels (Figure 1). Epitope-tagged (C-terminal) fragments of 42 and 21 kDa also increased during encystation. These are likely physiologically processed C-terminal fragments, as the antigens are prepared by trichloroacetic acid precipitation of live parasites in the presence of proteinase inhibitors to prevent degradation. Nonetheless, we cannot be certain that the native HCNCp processes the same way as epitope-tagged HCNCp.

**Figure 3:**
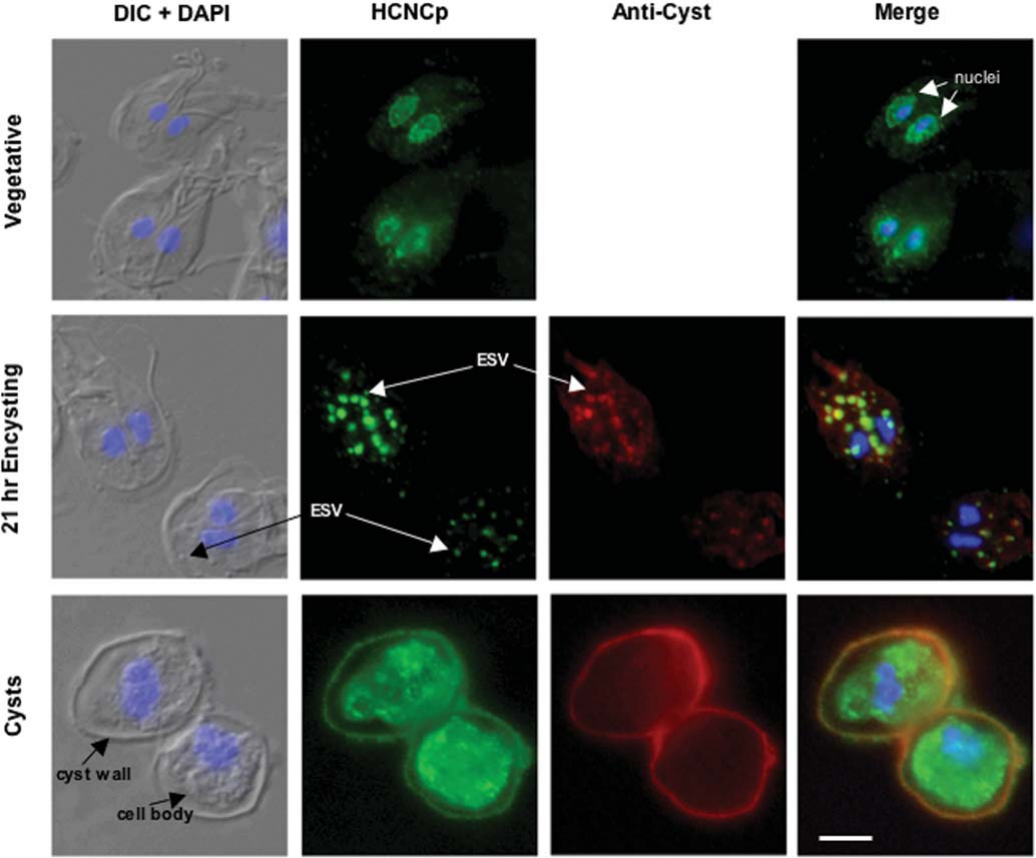
Traffic of HCNCp And Cyst Proteins During Growth And Differentiation Differential interference contrast (DIC) merged with DAPI images are shown to the left of each panel, HCNCp in green, anti-cyst proteins in red, and nucleic acid in blue (DAPI). In trophozoites, HCNCp localized to nuclei and nuclear envelope/ER. During encystation HCNCp co-localized with cyst proteins in encystation secretory vesicles (ESV) and to the cyst wall of water-resistant cysts. In mature cysts, most of the HCNCp was within the cell body. Scale bar is 5 µM.

In vegetative trophozoites, HCNCp was primarily localized to the nuclei (Figure 3), as shown by DAPI staining. Double staining with antibody to protein disulfide isomerase 2 (PDI2; [39]; data not shown) confirmed that some HCNCp was located to the nuclear envelope, which is the first cisterna of the ER. Encystation is not synchronous and we observed this nuclei/ER staining in some encysting cells (not shown). We found that during encystation, HCNCp traffics through the encystation secretory vesicles (ESV), which export CWPs. Double staining with rabbit anti-cyst antibody, which co-localized with cwp-1 and –2 to the ESV and cyst wall (not shown), showed co-localization of cyst proteins with HCNCp in ESV (Figure 3). Double staining also showed HCNCp in the wall of mature, water-resistant cysts. However, in contrast to the cyst proteins that were exclusively in the cyst wall, most of the HCNCp localized to the cell body of the cyst that becomes the excyzoite (Figure 3). We have not observed HCNCp in the plasmalemma of vegetative or encysting trophozoites, even though our epitope-tag contains the C-terminal predicted TM sequence.

### HCNCp is a Unique Non-Variant Cyst Protein

**Figure 4:**
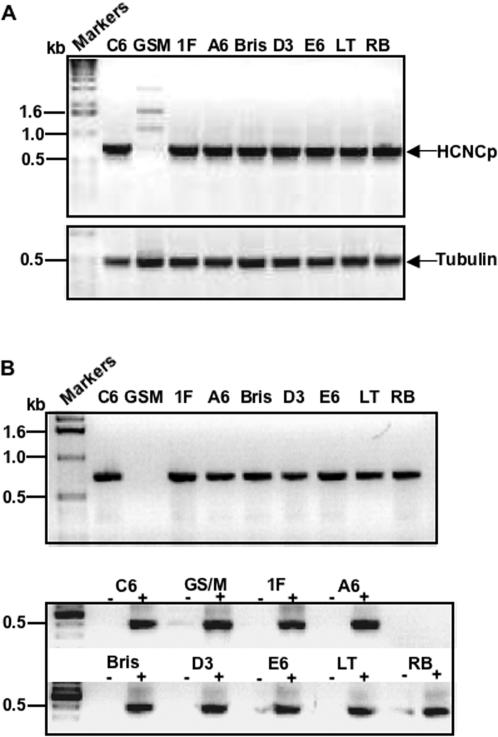
Presence Or Absence Of The HCNCp Gene And mRNA Transcripts In Trophozoites Of 9 *Giardia* Strains/Subclones (A) Genomic PCR found that the gene for HCNCp is present in all giardial isolates except for GS/M (upper panel). Lower panel shows tubulin controls for each isolate. (B) HCNCp mRNA transcripts were detected in all strains/clones tested except for the most divergent known *Giardia* strain infecting humans, GS/M (upper panel). Tubulin controls for rt-PCR without reverse transcriptase (“-“) or plus reverse transcriptase (“+”) are shown in the lower panel. Sizing markers are indicated by dashes to the left side of the figure in kb.

We searched the *G. lamblia* genome for sequences unique to HCNCp in order to develop HCNCp-specific internal PCR primers. We used these primers to detect the HCNCp gene and mRNA transcripts in different *Giardia* isolates. Isolate WB (clone C6) expresses the VSP called TSA 417 and 1F, A6, and E6 are subclones derived from WB C6 that do not express TSA 417 [24]. Bris, RB, and LT are unrelated human isolates, while D3 was isolated from a dog (American Type Culture Collection; ATCC). All are in Assemblage A [8]. We detected the HCNCp gene (Figure 4A) and transcript (Figure 4B) in trophozoites of all strains/subclones except GS/M, which belongs to Assemblage B and is considered the most distant *Giardia* strain infecting humans [40]. Other primer sets we tested also did not detect HCNCp in GS/M. The GS/M strain either does not have the gene or it is too dissimilar to be detected with the primer sets used. These data suggest that HCNCp is not a classical giardial VSP, but a new invariant cyst protein.

### Identification of other non-VSP cysteine-rich membrane proteins

We searched the *G. lamblia* genome for additional genes encoding a Type 1 integral membrane, high Cys containing proteins (HCMp) lacking the canonical VSP cytoplasmic tail sequence CRGKA (Table 1). Other search parameters were that the protein contain ≥400 amino acids, ≥10% Cys and ≥20 CxC and or CxxC. Of the 60 proteins we identified, none had specific sequence homology to HCNCp, but many contained the CxC motif that appears eight times in HCNCp, yet is rarely found in VSPs [16], [40]. Only one HCMp (ORF 17328) used the extended VSP polyadenylation signal [8], [18], while all others utilized the conventional *Giardia* polyadenylation signal. SAGE data was available for a number of these HCMp (Table 2), but only five had evidence of changes in expression during encystation. ORFs 16318 and 25816 were upregulated in 4–12 hr encystation, while ORFs 11309, 14017, and 15317 were approximately equal in abundance in trophozoites and during encystation, but nearly absent at 42 hr encystation. Each of these five proteins is a large, VSP-like protein with epidermal growth factor (EGF)-like motifs (Table 3). We also found 26 ORFs (18 were <400 aa) that were ≥10% Cys that had a single TM domain and no CRGKA tails, but they did not have ≥20 CxC/CxxC motifs (Table S1).

**Table 2:**
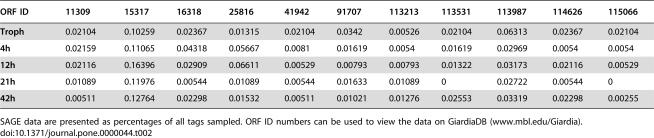
Available SAGE Data For The High Cysteine Membrane Proteins (HCMp) Outlined In Table 1

ORF ID	11309	15317	16318	25816	41942	91707	113213	113531	113987	114626	115066
**Troph**	0.02104	0.10259	0.02367	0.01315	0.02104	0.0342	0.00526	0.02104	0.06313	0.02367	0.02104
**4h**	0.02159	0.11065	0.04318	0.05667	0.0081	0.01619	0.0054	0.01619	0.02969	0.0054	0.0054
**12h**	0.02116	0.16396	0.02909	0.06611	0.00529	0.00793	0.00793	0.01322	0.03173	0.02116	0.00529
**21h**	0.01089	0.11976	0.00544	0.01089	0.00544	0.01633	0.01089	0	0.02722	0.00544	0
**42h**	0.00511	0.12764	0.02298	0.01532	0.00511	0.01021	0.01276	0.02553	0.03319	0.02298	0.00255

SAGE data are presented as percentages of all tags sampled. ORF ID numbers can be used to view the data on GiardiaDB (www.mbl.edu/Giardia).

**Table 3:**
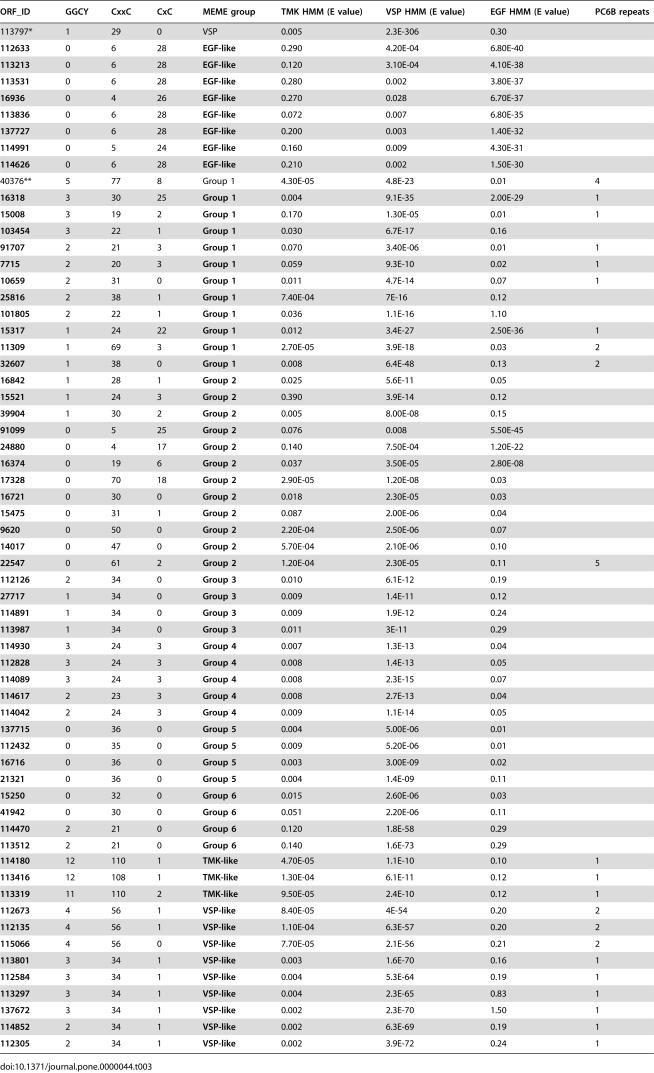
HCMp Categories And HMM Analyses

ORF_ID	GGCY	CxxC	CxC	MEME group	TMK HMM (E value)	VSP HMM (E value)	EGF HMM (E value)	PC6B repeats
113797*	1	29	0	VSP	0.005	2.3E-306	0.30	
**112633**	0	6	28	**EGF-like**	0.290	4.20E-04	6.80E-40	
**113213**	0	6	28	**EGF-like**	0.120	3.10E-04	4.10E-38	
**113531**	0	6	28	**EGF-like**	0.280	0.002	3.80E-37	
**16936**	0	4	26	**EGF-like**	0.270	0.028	6.70E-37	
**113836**	0	6	28	**EGF-like**	0.072	0.007	6.80E-35	
**137727**	0	6	28	**EGF-like**	0.200	0.003	1.40E-32	
**114991**	0	5	24	**EGF-like**	0.160	0.009	4.30E-31	
**114626**	0	6	28	**EGF-like**	0.210	0.002	1.50E-30	
40376**	5	77	8	Group 1	4.30E-05	4.8E-23	0.01	4
**16318**	3	30	25	**Group 1**	0.004	9.1E-35	2.00E-29	1
**15008**	3	19	2	**Group 1**	0.170	1.30E-05	0.01	1
**103454**	3	22	1	**Group 1**	0.030	6.7E-17	0.16	
**91707**	2	21	3	**Group 1**	0.070	3.40E-06	0.01	1
**7715**	2	20	3	**Group 1**	0.059	9.3E-10	0.02	1
**10659**	2	31	0	**Group 1**	0.011	4.7E-14	0.07	1
**25816**	2	38	1	**Group 1**	7.40E-04	7E-16	0.12	
**101805**	2	22	1	**Group 1**	0.036	1.1E-16	1.10	
**15317**	1	24	22	**Group 1**	0.012	3.4E-27	2.50E-36	1
**11309**	1	69	3	**Group 1**	2.70E-05	3.9E-18	0.03	2
**32607**	1	38	0	**Group 1**	0.008	6.4E-48	0.13	2
**16842**	1	28	1	**Group 2**	0.025	5.6E-11	0.05	
**15521**	1	24	3	**Group 2**	0.390	3.9E-14	0.12	
**39904**	1	30	2	**Group 2**	0.005	8.00E-08	0.15	
**91099**	0	5	25	**Group 2**	0.076	0.008	5.50E-45	
**24880**	0	4	17	**Group 2**	0.140	7.50E-04	1.20E-22	
**16374**	0	19	6	**Group 2**	0.037	3.50E-05	2.80E-08	
**17328**	0	70	18	**Group 2**	2.90E-05	1.20E-08	0.03	
**16721**	0	30	0	**Group 2**	0.018	2.30E-05	0.03	
**15475**	0	31	1	**Group 2**	0.087	2.00E-06	0.04	
**9620**	0	50	0	**Group 2**	2.20E-04	2.50E-06	0.07	
**14017**	0	47	0	**Group 2**	5.70E-04	2.10E-06	0.10	
**22547**	0	61	2	**Group 2**	1.20E-04	2.30E-05	0.11	5
**112126**	2	34	0	**Group 3**	0.010	6.1E-12	0.19	
**27717**	1	34	0	**Group 3**	0.009	1.4E-11	0.12	
**114891**	1	34	0	**Group 3**	0.009	1.9E-12	0.24	
**113987**	1	34	0	**Group 3**	0.011	3E-11	0.29	
**114930**	3	24	3	**Group 4**	0.007	1.3E-13	0.04	
**112828**	3	24	3	**Group 4**	0.008	1.4E-13	0.05	
**114089**	3	24	3	**Group 4**	0.008	2.3E-15	0.07	
**114617**	2	23	3	**Group 4**	0.008	2.7E-13	0.04	
**114042**	2	24	3	**Group 4**	0.009	1.1E-14	0.05	
**137715**	0	36	0	**Group 5**	0.004	5.00E-06	0.01	
**112432**	0	35	0	**Group 5**	0.009	5.20E-06	0.01	
**16716**	0	36	0	**Group 5**	0.003	3.00E-09	0.02	
**21321**	0	36	0	**Group 5**	0.004	1.4E-09	0.11	
**15250**	0	32	0	**Group 6**	0.015	2.60E-06	0.03	
**41942**	0	30	0	**Group 6**	0.051	2.20E-06	0.11	
**114470**	2	21	0	**Group 6**	0.120	1.8E-58	0.29	
**113512**	2	21	0	**Group 6**	0.140	1.6E-73	0.29	
**114180**	12	110	1	**TMK-like**	4.70E-05	1.1E-10	0.10	1
**113416**	12	108	1	**TMK-like**	1.30E-04	6.1E-11	0.12	1
**113319**	11	110	2	**TMK-like**	9.50E-05	2.4E-10	0.12	1
**112673**	4	56	1	**VSP-like**	8.40E-05	4E-54	0.20	2
**112135**	4	56	1	**VSP-like**	1.10E-04	6.3E-57	0.20	2
**115066**	4	56	0	**VSP-like**	7.70E-05	2.1E-56	0.21	2
**113801**	3	34	1	**VSP-like**	0.003	1.6E-70	0.16	1
**112584**	3	34	1	**VSP-like**	0.004	5.3E-64	0.19	1
**113297**	3	34	1	**VSP-like**	0.004	2.3E-65	0.83	1
**137672**	3	34	1	**VSP-like**	0.002	2.3E-70	1.50	1
**114852**	2	34	1	**VSP-like**	0.002	6.3E-69	0.19	1
**112305**	2	34	1	**VSP-like**	0.002	3.9E-72	0.24	1

We found that cysteine-rich (≥10%), Type 1 integral membrane proteins with ≥20 CxC and/or CxxC are absent or rare in most completed genomes (Table 4). For example, we found no representatives in the parasites *Toxoplasma gondii, Trypanosoma brucei, Plasmodium falciparum,* or *Cryptosporidium parvum* or in the yeast and plant model organisms *Saccharomyces cerevisiae, Arabidopsis thaliana* and *Chlamydomonas rheinhardtii*. In contrast, we found 6 HCMp's in the intestinal dwelling, cyst-forming human parasite *Entamoeba histolytica,* 4 each in *Caenorhabditis elegans* and *Drosophila melanogaster*, and 7 in humans. Surprisingly, the genome of the ciliated protozoan *Tetrahymena thermophila* has 30 HCMp. This number is still far fewer than *Giardia* if the genome size is considered. Based on predicted ORFs, *T. thermophila* has 5 times fewer HCMp than *Giardia* (Table 4). These 30 ORFs had high expectation values (from 0.0035 to 4.6e^−08^) to the TMK-like HMM from *E. histolytica* and half of them had between 1 and 25 PC6B motifs.

**Table 4:**
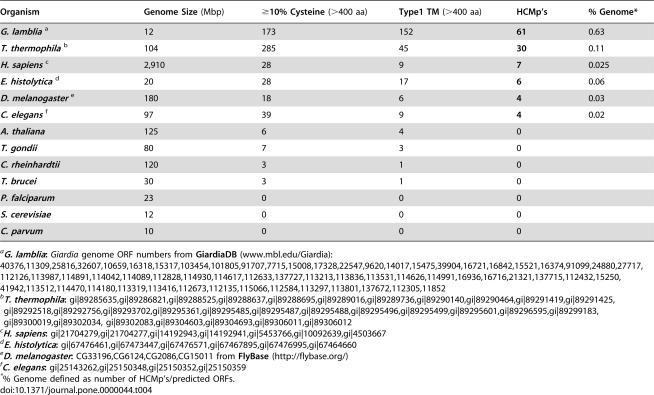
HCMp's In Model Organisms And Selected Parasite Genomes

Organism	Genome Size **(Mbp)**	≥10% Cysteine **(>400 aa)**	Type1 TM **(>400 aa)**	HCMp's	% Genome*
***G. lamblia*** a	12	173	152	**61**	0.63
***T. thermophila*** b	104	285	45	**30**	0.11
***H. sapiens*** c	2,910	28	9	**7**	0.025
***E. histolytica*** d	20	28	17	**6**	0.06
***D. melanogaster*** e	180	18	6	**4**	0.03
***C. elegans*** f	97	39	9	**4**	0.02
***A. thaliana***	125	6	4	0	
***T. gondii***	80	7	3	0	
***C. rheinhardtii***	120	3	1	0	
***T. brucei***	30	3	1	0	
***P. falciparum***	23	0	0	0	
***S. cerevisiae***	12	0	0	0	
***C. parvum***	10	0	0	0	

a ***G. lamblia*** **:** *Giardia* genome ORF numbers from **GiardiaDB** (www.mbl.edu/Giardia):

40376,11309,25816,32607,10659,16318,15317,103454,101805,91707,7715,15008,17328,22547,9620,14017,15475,39904,16721,16842,15521,16374,91099,24880,27717,112126,113987,114891,114042,114089,112828,114930,114617,112633,137727,113213,113836,113531,114626,114991,16936,16716,21321,137715,112432,15250,41942,113512,114470,114180,113319,113416,112673,112135,115066,112584,113297,113801,137672,112305,11852

b ***T. thermophila*** **:** gi|89285635,gi|89286821,gi|89288525,gi|89288637,gi|89288695,gi|89289016,gi|89289736,gi|89290140,gi|89290464,gi|89291419,gi|89291425,gi|89292518,gi|89292756,gi|89293702,gi|89295361,gi|89295485,gi|89295487,gi|89295488,gi|89295496,gi|89295499,gi|89295601,gi|89296595,gi|89299183,gi|89300019,gi|89302034, gi|89302083,gi|89304603,gi|89304693,gi|89306011,gi|89306012

c ***H. sapiens*** **:** gi|21704279,gi|21704277,gi|14192943,gi|14192941,gi|5453766,gi|10092639,gi|4503667

d ***E. histolytica*** **:** gi|67476461,gi|67473447,gi|67476571,gi|67467895,gi|67476995,gi|67464660

e ***D. melanogaster*** **:** CG33196,CG6124,CG2086,CG15011 from **FlyBase** (http://flybase.org/)

f ***C. elegans*** **:** gi|25143262,gi|25150348,gi|25150352,gi|25150359

<?ENTCHAR ast?> % Genome defined as number of HCMp's/predicted ORFs.

### Bioinformatic analyses of HCMp

36 of the giardial HCMp are annotated as VSPs in the *Giardia* Genome Database (www.mbl.edu/Giardia [41]), nine as notch/EGF receptor-like [42], and 16 as *E. histolytica* receptor kinase-like proteins [43]. We used comparative motif analyses with the programs MEME [44] and MAST [45] to determine whether any of the 61 *G. lamblia* HCMp (Table 1) contain repeats or known motifs or might be related to each other. Seven of the nine similarity groups contained regular repeat patterns (Figures S1 and S2). In addition to the repeated motifs, three groups (called VSP-like, EGF-like, and TMK-like) contained recognizable motif signatures. The proteins we placed in the VSP-like group had expectation values of e^−54^ to e^−72^ against the VSP domain HMM of the Pfam database (PF03302), while TSA 417 was e^−308^ (Table 3). The EGF-like group had high similarities to the EGF functional domain (PF00008). The TMK-like group scored highly to a HMM constructed using alignments from the extracellular domains from 6 of 8 *E. histolytica* transmembrane kinases [43]. In addition, some members of the other groups had fewer instances of these motifs. Several members of the TMK-like, VSP-like, and Group 1, including HCNCp, also contained 1 or more PC6B domains (Table 3), which have been implicated in the possible stabilization or intracellular localization of a Kex-2 like endoproteinase [46].

## Discussion

The sequence of HCNCp is strikingly similar to the VSPs in many respects, however its expression and trafficking largely resemble that of the CWPs. Moreover, we discovered that the giardial genome contains a total of 60 additional high cysteine Type 1 integral membrane proteins (HCMp) that resemble HCNCp, and do not appear to be VSPs. The giardial isolate (“WB, clone C6”) used for genome analyses has about 150 VSPs in its repertoire. VSPs change under selection by antibodies *in vitro* and *in vivo* [11], [12]. However, they also switch spontaneously [25], [47]. VSP expression may also be influenced by physiological conditions, such as intestinal proteases or growth vs. differentiation [24], [32] and may help in adaptation to growth in different host species [48]. In contrast, HCNCp is not variable, as it was expressed in all the Assemblage A isolates and subclones we tested.

VSPs may differ greatly in length from about 20 to 200 kDa, frequently because of different numbers of repeated sequences [49], [50]. They often belong to small families whose genes are recognized by the same probes or encode proteins with cross-reactive epitopes. Many VSP sequences have been used as to define different genetic assemblages [7], [40], [51]. Despite their variability, the VSPs have strikingly constant features. They are all highly (∼11–12%) cysteine-rich Type I integral membrane proteins, with a large proportion of the Cys residues in “CxxC” tetrapeptide motif, where x can be many amino acids [16]. However, the CxC motif is very rare in VSPs [7], [16]. CC sequences are rarely found in VSPs or HCMp. VSPs have a conventional N-terminal signal peptide and least one GGCY tetrapeptide motif near the C-terminus. Many have a Zn finger motif. VSPs are characterized by an extremely conserved C-terminal membrane spanning sequence and short hydrophilic cytoplasmic anchor sequence, CRGKA. The Cys is a substrate for palmitoylation [52], which may be a means to regulate cellular signaling. Virtually all of the HCMp also have Cys residues in their tails that may act as a substrate for this modification, although *Giardia* has relatively few palmitoylated proteins [53], [54]. While most giardial genes have a typical consensus AGTUAAY polyadenylation signal, that of the VSPs is 15 nucleotides [8], [18]. Most importantly, VSPs traffic to the plasma membrane via the ER in a constitutive secretory pathway [7], [32], [35]. These features may be considered diagnostic of VSPs.

The three known cyst wall structural proteins (CWPs) are similar to each other with a signal peptide and leucine-rich repeats (LRR), but no TM regions [28]. The 14 Cys residues they share are positionally conserved and they do not appear to vary among isolates. Expression of the CWPs is greatly upregulated in encystation and they are transported to the nascent, extracellular cyst wall by novel encystation secretory vesicles (ESV) that are specifically assembled during encystation [34].

Although *Giardia* was reported to lack a conventional Golgi apparatus [55], we showed earlier that it could sort proteins to constitutive and regulated secretory pathways [24], [34]. Specifically, in vegetative cells, the VSP TSA 417 is targeted to the plasmalemma via the constitutive pathway [32]. During encystation, TSA 417 co-localizes with CWPs in the nuclear envelope and the ER, but does not enter the ESVs or traffic to the cyst wall. Conversely, CWPs are sequestered in the ESVs for transport to the cyst wall, but not found in the plasmalemma [26]–[28], [34]. Rather than a discrete targeting motif, most of the LRR regions of the CWPs seem to be required for entry into the ESV and incorporation into the cyst wall [28], [34], [56]. Hehl's group also reported that chimeras containing the LRRs from a CWP [56] and the VSP [57] transmembrane region and tail localized to the plasmalemma in pre-encysting cells lacking ESV. However, during encystation, the chimera was targeted to the cyst wall via ESVs. This group has also localized homologs of Golgi proteins to ESVs [58], [59] and proposes that they have post-ER Golgi-like sorting function.

In vegetatively growing cells, HCNCp localizes largely to the nuclei and nuclear envelope, as it co-localizes with DAPI staining and PDI-2 (Figure 3). Thus, it is within the innermost cisterna of the ER and in the constitutive protein secretory pathway [32]. In contrast to the VSPs, it is not in the more distal ER or the plasmalemma. The implications of HCNCp within the nuclei remain to be elucidated. In encysting cells, HCNCp clearly co-localizes with cyst proteins within the ESV, which demarcate the regulated secretory pathway [34]. In mature cysts, virtually all anti-cyst epitopes are in the extracellular cyst wall and little is detected within the cell body. However, only a fraction of HCNCp is in the cyst wall and most appears to be in vesicular structures throughout the cell body (Figure 3). We cannot tell if these are remnant ESVs that have discharged the CWPs, but retain at minimum the C-terminus of HCNCp. This is the first observation of a protein that localizes to both the cyst wall and cell body. Since the cell body is the precursor of the excyzoite, HCNCp may have a function during or after excystation. Therefore, HCNCp appears to follow a novel protein trafficking pathway, unlike those previously characterized in *Giardia*. We trace HCNCp with a C-terminal 6-amino acid epitope-tag. Thus, even the smallest fragment detected (∼21 kDa) contains the hydrophobic putative TM region. Further studies are needed to determine the fate of the N-terminus of HCNCp and the function, if any of the proteolytic processing.

In addition to HCNCp, another cyst protein is proteolytically processed during encystation. Lujan et al (1995) [26] reported that CWP2 can be proteolytically cleaved from a 39 kDa protein to 26 kDa by an encystation specific cysteine proteinase (ESCP) found in the lysosomal compartment [60]. We have not determined whether ESCP or other proteinases can cleave HCNCp, nor do we know if it can be self-cleaving. We have found a putative subtilisin-type proprotein convertase (SPC) that is also upregulated in encystation and localizes to the ESV (B. J. Davids unpublished data). Interestingly, there is an SPC cleavage site in HCNCp that would produce a C-terminal epitope-tagged fragment of v56 kDa, which may be one of the minor fragments seen in our immunoblot of cysts (Figure 2). The VSP TSA 417 also undergoes endogenous proteolysis [16], but the fragments are held together on the cell surface by disulfide bonding.

Although it is strikingly cysteine-rich, with 77 CxxC motifs, HCNCp clearly differs from the VSPs, especially in its C-terminus. HCNCp also has 6 CxC motifs while TSA 417, like most other VSPs, has none. We asked whether HCNCp is unique or resembles other proteins in *Giardia*. Genome-wide analyses revealed 173 giardial proteins of ≥400 amino acids with ≥10% Cys. 152 of these are Type 1 integral membrane proteins. Of these, 89 have a VSP CRKGA tail and have not been considered in our analyses. Aside from the VSPs, 61 ORFs including HCNCp have ≥20 instances of CxxC/CxC and satisfy all our criteria for a putative HCMp. The HCMp are difficult to classify and differ in various parameters, although they form small clusters with similar membrane spanning and or cytoplasmic sequences (Table 1), and the occurrence of GGCY motifs (Table 3). While not all groups had GGCY motifs, HCNCp had 5 and others within “group 1” had between 1 and 3. The significance of this motif in VSPs has not been fully examined, although Nash [7] reports that mutations in GGCY result in loss of VSP surface localization. We have not seen HCNCp localized at the surface membranes of trophozoites or encysting trophozoites, but it may have an important role at the surface of excyzoites during excystation.

The HCMp also differ from each other in whether they have a preponderance of CxxC or CxC motifs. The latter are very rare in VSPs [7]. All have some CxxC motifs and many have no CxC, but others have from 1 to 28 CxC sequences. From a functional point of view [61], CxxC motifs are well-known in the active sites of redox proteins like PDIs and thioredoxins. The identity of the two intervening amino acids strongly affects the E^0′^ or redox potential. We showed earlier that in TSA 417 [16], all detectable Cys residues were in intramolecular disulfide bonds that were extremely resistant to reduction in the native protein. However, the vicinal disulfide bonds of three amino acid CxC rings are much less stable than those of four-member CxxC rings. The functional implications of the prevalence of CxxC vs CxC motifs in HCMp remain to be determined.

We asked whether extremely cysteine-rich membrane proteins are common in other organisms, especially those that live in a microaerophilic environment similar to that of *Giardia*. *E. histolytica* is a mucosal dwelling protozoan that also differentiates into cysts. Both parasites require high concentrations of Cys for growth in culture [62], [63]. *E. histolytica* has 17 Type 1 integral membrane proteins with ≥10% Cys, but only 6 of these proteins have CxxC/CxC sequences that would qualify them as HCMp [64]. Four of the other genomes we examined had fewer HCMp than *Giardia* although *T. thermophila* has 30 (Table 4). However, only 0.11% of the macronuclear *T. thermophila* genome is dedicated to HCMp [65], compared to 0.63% for *Giardia*. Currently, these two protozoans appear to have more HCMp than other organisms, although analyses of additional genomes could certainly reveal others. Genetic manipulations are not possible in *Giardia*, which is tetraploid [66]. However, reverse genetics [67] in *T. thermophila* could provide clues or hypotheses on functions of HCMps.

Global comparisons of the HCMp are challenging as they group differently depending on the parameter used. Within the giardial HCMp, recognizable motifs (Figures S1 and S2) enabled us to classify them into subgroups (Table 3). These subcategories may represent specific classes of HCMp with various functions. It will be important to characterize different members of each MEME category to determine if their group similarity can predict possible expression, localization, and function during different stages of giardial differentiation. Presently, we can say that HCNCp has structural characteristics like that of VSPs, but is regulated and expressed similar to a CWP. It is interesting that the known giardial surface proteins are all cysteine-rich. This may help explain the high requirement of *Giardia* for Cys for growth and survival [62], [68]. HCNCp is a member of a large group of cysteine-rich, non-VSP, Type 1 integral membrane proteins, which are rare to absent in the majority of genomes examined. The exact functions of HCNCp and other HCMp, their evolutionary origins, and their overall importance to the biology of *G. lamblia* remain to be determined.

## Materials and Methods

### Giardial isolates, cultivation and encystation

We used isolate WB clone C6 (ATCC 50803; [6]; human, Afghanistan) unless otherwise indicated. We tested expression of HCNCp in trophozoites of WB C6 subclones (1F, E6 [24]), A6 (ATCC 50583; [69]), and strains LT ([70]; human, Puerto Rico), RB (isolated from a patient infected in Turkey), D3 (ATCC 203334, isolated from a dog Calgary, Alberta, Canada, 1986), Bris ([71], human, Australia), and GS clone H7 (GS/M; ATCC 50581; [72], human, Alaska). Parasites were grown in modified TYI-S33 medium and encysted as described [37].

### Production of polyclonal anti-cyst antibody

Polyclonal antiserum against *in vitro* purified water-resistant cysts was raised in rabbits as described [73].

### mRNA expression patterns

Serial Analysis of Gene Expression (SAGE) libraries were constructed using ∼10 µg of total RNA isolated from TRIzol (Invitrogen, Carlsbad, CA) extractions of trophozoites and encysting cells (4, 12, 21, 42 hr) following the I-SAGE protocol (Invitrogen). Recombinant pZero1 clones produced by SAGE were purified using GeneMachines RevPrep Orbit (San Carlos, CA) and were sequenced on an ABI 3730xl DNA sequencer (Applied Biosystems, Foster City, CA). Sequences collected were analyzed with software created at the MBL specifically for *Giardia* SAGE analyses. The SAGE software extracts ditag sequences from the ABI 3730xl results according to the SAGE sequence grammar, parses out individual SAGE tags, excludes tags with sequence ambiguities, and reduces all SAGE tags to a look-up table of unique SAGE tag sequences and their observed frequencies among all of the *Giardia* SAGE libraries. SAGE tags not found more than once in at least one SAGE library were excluded from analyses as putative sequencing error, unless the tag sequence had a perfect match to available genome data. The unique tag sequences were mapped to all available *Giardia* “giardia14” genome assembly contigs (www.mbl.edu/Giardia) to determine the identity of expressed genes. The 15 bp SAGE tags were mapped to the assembly based on exact sequence matches.

HCNCp mRNA expression was verified by Northern analysis. 10 µg of total RNA was extracted from trophozoites and encysting cells as above and separated on a denaturing RNA gel, transferred to a ZetaProbe filter (Bio-Rad Laboratories, Hercules, CA), and hybridized as described [28]. The strand-specific 244 bp probe for HCNCp was made using primers “Nprobe-for” (5′GCAGCATTCCTACCCGTTAT3′) and “Nprobe-rev” (5′TCCAAACATTGCGTTGATTC3′) in combination with the labeling kit Prime-it II (Stratagene, La Jolla, CA). The probed and washed filter was imaged on a Storm 860 phosphoimager (Molecular Dynamics, Sunnyvale, CA).

### Selectable transformation and epitope tagging

The promoter and coding sequences for HCNCp were amplified from 200 ng of genomic DNA using the forward primer “HCNCp-for” (5′-ACAAGCTTTCACAGGATGTGGACGAT-3′) and the reverse primer “HCNCp-rev” (5′-AAATGGGCCCCACAGCTTTGCTCCTAC-3′) and the GeneAmp XL PCR kit (Applied Biosystems, Branchburg, NJ) by standard conditions at a final concentration of 1.2 mM Mg(OAc)_2_. Conditions for PCR were an initial denaturation at 95°C for 5 min, 4 cycles of: 95°C 50 sec, 50°C 1 min, 72°C 6 min; 31 cycles of: 95°C 50 sec, 55°C 1 min, 72°C 6 min; and a final extension for 10 min at 72°C (MJ Research, Waltham, MA). The ∼4 kb product was gel purified using QIAquick gel extraction kit (Qiagen; Valencia, CA) and approximately 1 µg of each PCR product or AU1-epitope tagging *Giardia* vector [74] were first digested with Apa1 for 1 hr 37°C, cleaned with QIAquick for PCR products, digested with HindIII for 1 hr 37°C, and gel purified using QIAquick gel extraction kit. The vector was de-phosphorylated using shrimp alkaline phosphatase (Promega; Madison, WI) by standard methods and purified using QIAquick. The digested vector and PCR fragments were ligated (T4 DNA ligase; Roche, Nutley, NJ) overnight at 4°C, transformed into JM109 competent cells (Promega), and plasmids screened by size for automated sequencing of clones containing full length HCNCp (GenBank accession number DQ144994). WB clone C6 trophozoites were electroporated with 50 µg of plasmid containing HCNCp and were selected and maintained by puromycin resistance [39]. Recombinant clones were purified using GeneMachines RevPrep Orbit, sequenced on an ABI 3730xl DNA sequencer (Applied Biosystems), and assembled using the computer program PHRAP [75].

### Expression of HCNCp

Trophozoite, encysting cells, and cyst antigens from *Giardia* stably expressing the AU1-tagged HCNCp gene or non-transfected control cells were processed for Western analysis [76]. Because of the large size of HCNCp, transfer buffer contained 20% MeOH/0.1% SDS. Blotted proteins were blocked overnight in PBST (PBS+0.05% Tween 20) containing 5% milk, probed in rabbit anti-AU1 antibody (1/5,000 in PBST/0.1% milk; Bethyl Laboratories, Montgomery, TX) or antibody to the constitutive protein taglin (1/700 in PBST/0.1% milk; loading control; [77]) for 1.5 hr, washed 3× over 30 min in PBST, incubated in Zymax goat anti-rabbit (1/8,000; Zymed Laboratories/Invitrogen) or Zymax goat anti-mouse (1/5,000; Zymed Laboratories/Invitrogen) antibodies for 1 hr, washed 3× in PBST over 30 min, and developed in ECL (Amersham Biosciences, Piscataway, NJ).

### Immunolocalization of HCNCp and CWP

Transfected parasites stably expressing AU1-tagged HCNCp or non-transfected control cells were prepared for immunofluorescence assay (IFA) by methods similar to Abel et al., 2001 [21]. Giardial trophozoites or encysting cells were allowed to attach to glass coverslips coated with 0.1% polyethylenimine (Sigma, St. Louis, MO) in PBS on a slide warmer at 37°C. Water-resistant cysts were allowed to dry onto the coverslip and then placed in IFA block as described below. Attached cells were fixed in 100% methanol (−20°C) for 10 min, dried, and stored overnight at room temperature or permeabilized immediately in 0.5% Triton-X 100 for 10 min, and placed in IFA block (10% goat serum, 1% glycerol, 0.1% bovine serum albumin, 0.1% fish gelatin, and 0.04% sodium azide in PBS) for 1 hr. Cells were incubated for 1 hr in primary antibody (anti-AU1 mAb diluted 1/500 in IFA block; Covance, Berkeley, CA; or rabbit polyclonal anti-cyst (1/250 in IFA block)), washed 4× 5 min in PBS, and incubated in goat anti-mouse FITC-labeled (1/100 in IFA block; Pierce Biotechnology, Inc., Rockford, IL) or goat-anti rabbit TRITC-labeled (1/100 Zymed Laboratories/Invitrogen) secondary antibodies for 1 hr, washed 4× 5 min in PBS, post-fixed in 4% PFA for 7 min, rinsed 2× in PBS, and mounted with ProLong Gold with DAPI (Invitrogen). Cells were screened on an E800 Nikon (New York City, NY) microscope with an EXFO (Vanier, Canada) X-cite fluorescent 120W metal halide illuminator and imaged with a DMX 1200F Nikon fluorescence sensitive digital camera.

### RtPCR and genomic PCR

cDNA was prepared directly from 10^4^ log-phase trophozoites (SuperScript^TM^ III CellsDirect cDNA synthesis system, Invitrogen). HCNCp-specific internal primers were: 5′-GGAAAACGGTGACCTTATTGTG-3′ (forward) and 5′-GTCTACCAGGACTGAAAGTAT-3′ (reverse); and control primers for alpha-2-tubulin were: 5′-CGTTCAACACGTTCTTCTCG-3′ (forward) and 5′-ATGTCGTACATGGCCTCGTT-3′ (reverse). 3 µl of the cDNA template in combination with 0.2 mM dNTP mix, 2 mM MgSO4, 0.25 µM each primer, and 1 U Platinum Taq high fidelity polymerase (Invitrogen) was used in a standard PCR reaction. Conditions for PCR were an initial denaturation at 95°C for 3 min, 36 cycles of: 95°C 1 min, 50°C 1 min, 72°C 2 min, and a final extension for 7 min at 72°C.

Genomic DNA was prepared from all strains and subclones using DNA STAT-60^TM^ (Tel-Test, Inc., Friendswood, TX) by manufacturer protocols. Conditions for genomic PCR were as described above using ∼200 ng of DNA as template.

### Bioinformatic Analyses

A profile Hidden Markov Model (HMM) of the *Entamoeba histolytica* transmembrane kinase (TMK) extracellular domain [43] was created with HMMER version 2.3.2 [78]. The HMM's for VSP (PF03302) and EGF (PF00008) were from the Pfam database [79]. Conservative nucleotide or amino acid motifs were detected using Multiple Em for Motif Elicitation (MEME) [44] and Motif Alignment & Search Tool (MAST) [45], predication of signal peptides using SignalP 3.0 [80], and prediction of transmembrane domains using transmembrane Hidden Markov Model (TMHMM) 2.0 [81]. A custom PERL script was used to search genomes for cysteine-rich protein sequences containing CxC and/or CxxC motifs (A. G. McArthur). The PC6B motifs present in HCMp's were found using a regular expression constructed from the published consensus sequence: Cx_2_Cx_3_Cx_2_Cx_5-7_Cx_2_Cx_10-15_Cx_3_C [46].

## Supporting Information

Table S1 Cysteine-Rich Proteins Not Classified As HCMp Because They Are Too Short And/OR Have < 20 CxxC + CxC's
(0.04 MB PDF)

Figure S1 Diagram Of MEME/MAST Analyses Of The 9 HCMp Groupings
(0.29 MB PDF)

Figure S2 Motif Sequences Found By MEME Analysis For The 9 HCMp Groupings
(0.06 MB PDF)
