# Supplementary material for: A New Family of Giardial Cysteine-Rich Non-VSP Protein Genes and a Novel Cyst Protein
Source: PLoS One. 2006 Dec 20;1(1):e44. doi: 10.1371/journal.pone.0000044 (PMC1762436; doi:10.1371/journal.pone.0000044)
Supplement: Table S1 — Cysteine-Rich Proteins Not Classified As HCMp Because They Are Too Short And/OR Have < 20 CxxC + CxC's (0.04 MB PDF) [file pone.0000044.s001.pdf]

Supporting Table 1. Cysteine-Rich Proteins Not Classified As HCMp Because They Are Too Short And/Or Have ≤ 20 CxxC + CxC's

**ORF ID**	**% Cysteine**	**Length** (aa)	**CxxC**	**CxC**	**GGCY**
**34777**	12.5	40	0	1	0
**111877**	10.9	55	1	0	0
**25013**	14.3	63	3	3	0
**32701**	10	100	2	1	0
**35985**	11.3	115	4	2	0
**114888**	11.7	120	6	0	0
**98126**	10.6	123	1	1	0
**103943**	10.3	185	8	0	0
**28057**	11.5	191	10	0	0
**17212**	10.7	206	0	5	0
**9003**	11.3	212	11	0	0
**102180**	10.7	224	5	2	0
**112604**	10.7	224	5	2	0
**14324**	10.9	239	6	2	0
**14783**	11.7	308	12	0	1
**87706**	11.8	348	17	0	0
**14791**	12.6	350	18	0	0
**101589**	12.5	393	16	0	1
**115158**	10.13	454	15	0	1
**17380**	12.3	511	16	3	1
**6372**	12.4	533	1	10	0
**101832**	10.3	536	14	2	3
**114161**	11.2	556	1	10	0
**137732**	10.91	614	1	11	0
**94003**	11.2	677	2	11	0
**25238**	12.12	685	11	7	0

ORF ID numbers can be used to view data at GiardiaDB (www.mbl.edu/Giardia).
