# Supplementary material for: A New Family of Giardial Cysteine-Rich Non-VSP Protein Genes and a Novel Cyst Protein
Source: PLoS One. 2006 Dec 20;1(1):e44. doi: 10.1371/journal.pone.0000044 (PMC1762436; doi:10.1371/journal.pone.0000044)
Supplement: Figure S1 — Diagram Of MEME/MAST Analyses Of The 9 HCMp Groupings (0.29 MB PDF) [file pone.0000044.s002.pdf]

Supporting Figure 1. Diagram Of MEME/MAST Analyses Of The 9 HCMp Groupings

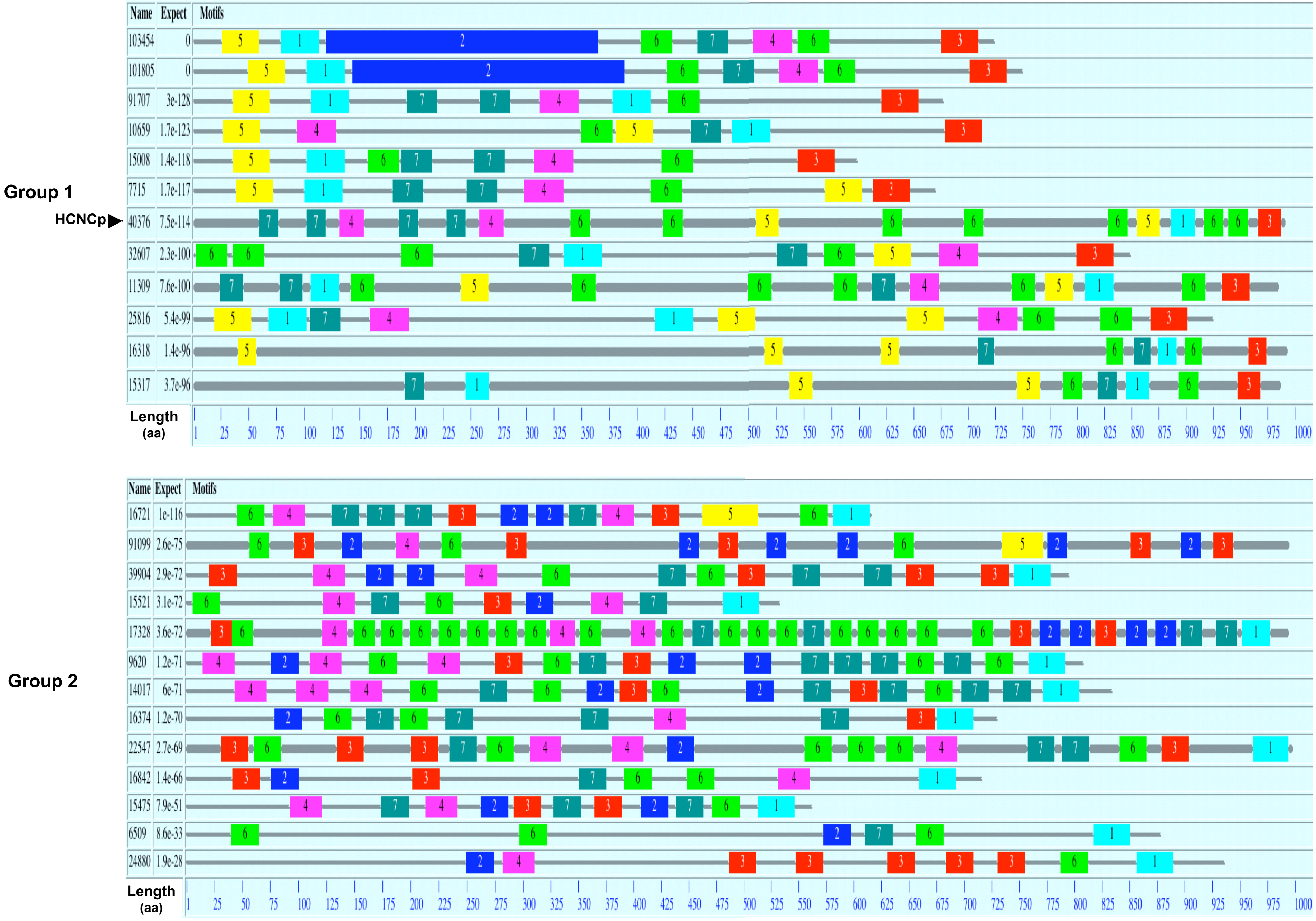

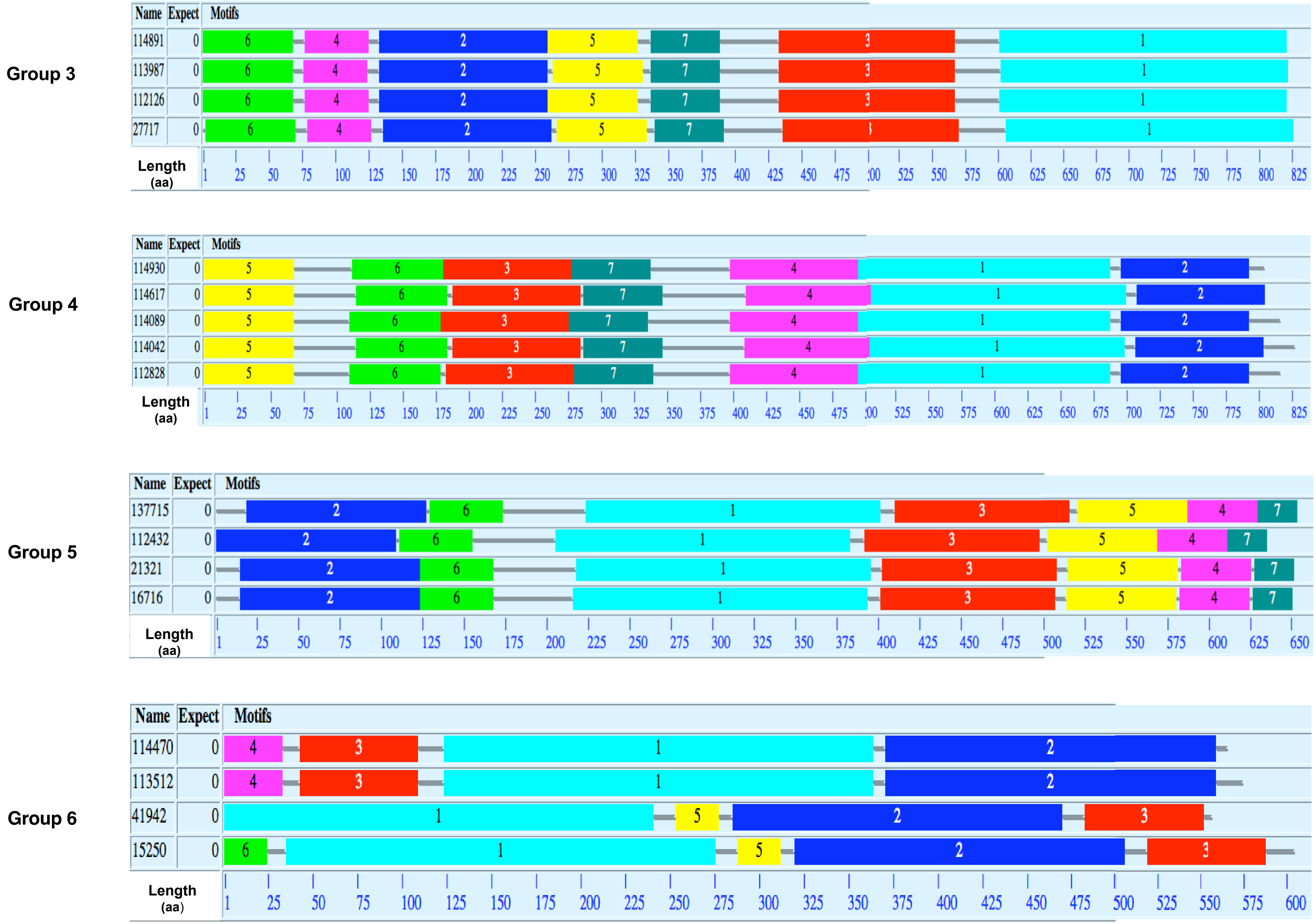
Group 3
Name Expect Motifs
114891 0
113987 0
112126 0
27717 0
Length (aa)
Group 4
Name Expect Motifs
114930 0
114617 0
114089 0
114042 0
112828 0
Length (aa)
Group 5
Name Expect Motifs
137715 0
112432 0
21321 0
16716 0
Length (aa)
Group 6
Name Expect Motifs
114470 0
113512 0
41942 0
15250 0
Length (aa)

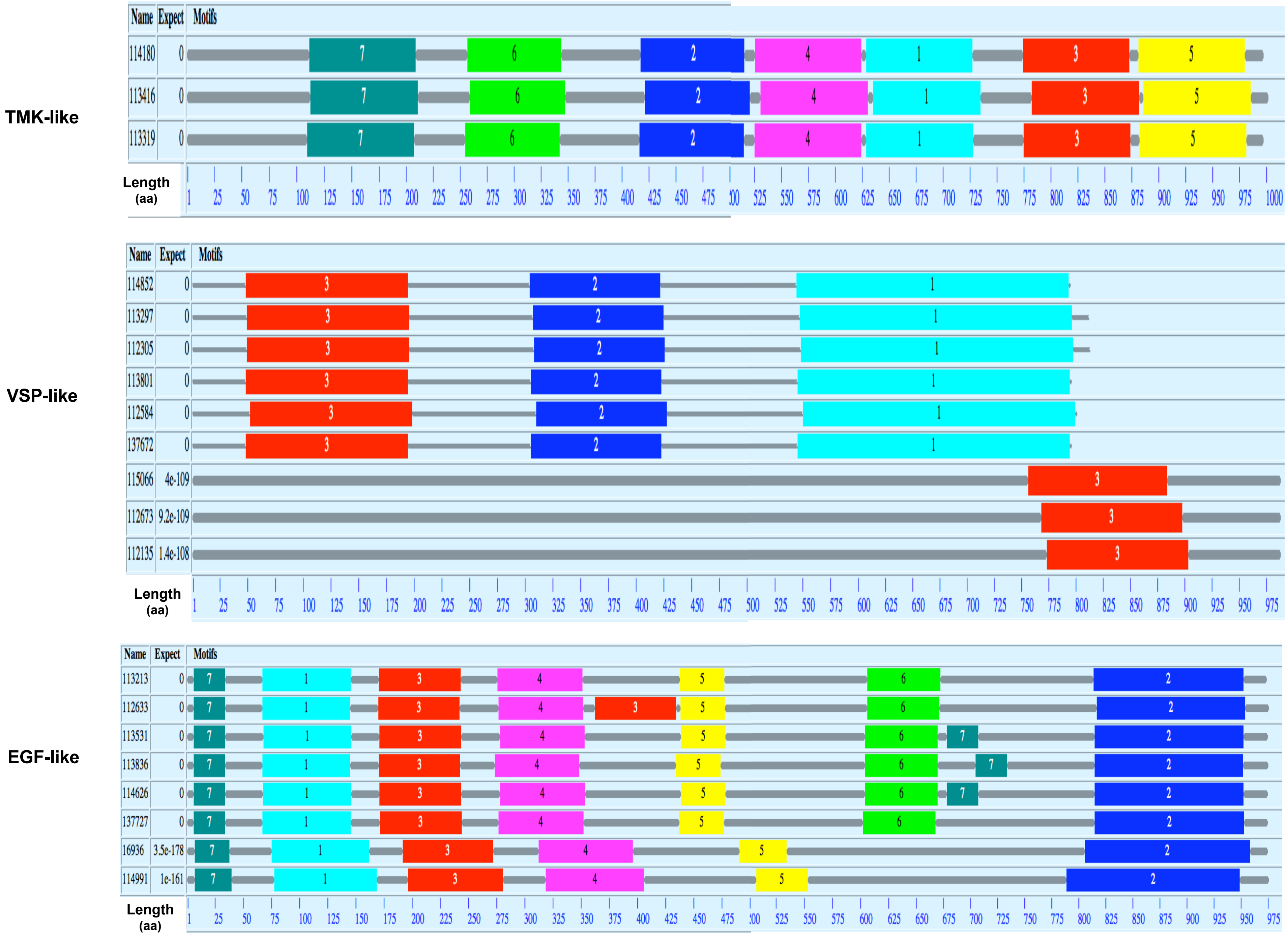

TMK-like
VSP-like
EGF-like
Name
Expect
Motifs
114180
113416
113319
114852
113297
112305
113801
112584
137672
115066
112673
112135
113213
112633
113531
113836
114626
137727
16936
114991
0
4e-109
9.2e-109
1.4e-108
3.5e-178
1e-161
Length (aa)
