# Supplementary material for: A New Family of Giardial Cysteine-Rich Non-VSP Protein Genes and a Novel Cyst Protein
Source: PLoS One. 2006 Dec 20;1(1):e44. doi: 10.1371/journal.pone.0000044 (PMC1762436; doi:10.1371/journal.pone.0000044)
Supplement: Figure S2 — Motif Sequences Found By MEME Analysis For The 9 HCMp Groupings (0.06 MB PDF) [file pone.0000044.s003.pdf]

Supporting Figure 2. Motif Sequences Found By MEME Analysis For The 9 HCMP Groupings

MEME group	MEME motif ID	MEME motif length (aa)	MEME motif sequence
**Group 1**	1	35	CTQCKDGYFLFMGGCYKIDRWPGNHVCKKAESGMC
**Group 1**	2	250	KFLFTNPDDSAAERCILCYDNVGFGSYKGVDGCRYCLPPLTGETSAECNWCQNEKYGPIDGVCKEPGRHLCAD<br>GACSNCYMTHIQHNGGCYTKTETVAQKICTTENQFQVINITACKKCAIKGEVPMDGRCIKGLLEPKCNPYPNTDG<br>SCTDCKKGGVNYDTFLFNEGCYNMSDYIGSQICSKVNERAECELWNIGDYGVFKPPDDHMAHPCSDTSARGIQ<br>GCSRCRYNNEEKKPICYDCEYDTLAFDRM
**Group 1**	3	34	LSAMAIIGIAIAVMVVVGALVGFLVWWFLFRRRR
**Group 1**	4	36	FLFMGGCYNINKYPGRFICKWAFDGKCTECMEHYGY
**Group 1**	5	34	QMDRHHMKCVMFVRGTEYCSQCRAGHVPINGICV
**Group 1**	6	29	CRACHENCATCSGPQADQCLSCMPGKYWK
**Group 1**	7	28	QFEINGYHYCSKCKDQSEFPHNGICKPH
**Group 2**	1	33	WMSMWMIVGISVFVIMLAAIIVWIIWWFVKRRK
**Group 2**	2	25	TCTRCAEGYFLWDGKCYPCEMIPGK
**Group 2**	3	25	YEHEEYCLKCMPGYYMINGRCVHAN
**Group 2**	4	29	CKIRNCVKCHDDKQMCEACKENFYLEKDG
**Group 2**	5	51	HRKRLFGRWLQQFQAKRADSQPANRREDWEKELQGQQQYYREADIRVNNVR
**Group 2**	6	25	MCKECNTDNGQQCESCHEGYYLDPT
**Group 2**	7	25	NCKQRDSATTCVECEPGYFLQPSRK
**Group 3**	1	220	GAKNVVCQKCEDGYLKEHDQNKVVCEPPPPECTVELCAICVEDDPNRCEHCTAGAYLCPRTAACTKDCASCGG<br>SMYPDEDLGECQPCDIANCRVCATGLKCESCAENAVPISPDVYHTACMGCSDTGGMDGWVGLAGCTTCELTDK<br>GGGSVNCLDPGYYRRGGLGGGAIAAIVIVVLLVLAVAGFLVWWFVFRRGGRPKRGAKYTSLMRGESYDYRQSLI
**Group 3**	2	129	GNTCDKGVCTTCGNGYFFHYGSCYKFDGSPGSIVCPNQTDTTGSVCGSCGSKFVKNPSASSSATSCLACYGLD<br>GTLFCERCRMNVLTSADVKPTLCTQCKIGYVPVDGRCISNYSALAALAGCSDPRGN
**Group 3**	3	137	EPPEDNACPQALPPATPEGCAVQGCQACSKDSATTCDACFEPLIMKEDKTKCFGSCLALGPGYYNNSGICAPCV<br>ENCLSCVDGTSCSQCAEGYYLEVSGADSSTGACKPCAAGCSTCNDAGDSLCSTCLFGYYIAFE
**Group 3**	4	49	CIRDFLFYRGGCYRTASLINAICKEKHIVEEGMYCGACAIEGDVPIDGV
**Group 3**	5	69	GLCEACSSGFMLFYGSCYSVDGPLAQAICKAEDQIYLNGRVFCKRCAQPDHYPINGLCIAQKGDHTDEH
**Group 3**	6	69	MRVLILLLLHGIFSECTQDSDCGGATGVCNVTIGGKKYCSKCDGARVPVNGDCRLLSLSRSICIVPTGS
**Group 3**	7	53	DTKQTADDTPEFFLFYNGCYDINHPIGKLICQEVSNGACSTCNSNESSNIIAN
**Group 4**	1	193	YPFNGVCIPNTQRDAYCTSTANGACTTCSGAAFLMNGGCYTTEHYPGSTICDKQSNGKCTTTKKGYWISPDGKL<br>LECDPTCLACTAPGPGRCTRCPSDKLLKRASGAATGSCVDPGACVDGYYADGDACLPCATPGCKTCGHASFCT<br>ECAGELFVSLDGQSCLEECTGDKVVGEVPGGVRRCWCERGFLPALD
**Group 4**	2	97	ECPPDMPWCAACDESGRCLLCVTSGHNVQVDQRTCAEGCGARASSNQGVCMCELDAVLTKGICVPAKELARK<br>RMAAIAGGTVAGVIVIGVLVGFLCW
**Group 4**	3	97	YTKQDRHFYPIDYQCQAKGLHNCVNGYCTSCYKTHLFYEYGCYSRHSSTGFAICAEANQYELDNVTICKECVNT<br>SHAPKNGACILIKENPSIMEFSD
**Group 4**	4	97	VEEGSESKEVSCRCVCMPTFYNSSGTCTPCTDSCAVCKDGTPTGCQQCSPGKILNFSIVSSENADCVDQCSVG<br>DECAECGITIDGSRYCTRCKDANM
**Group 4**	5	68	MLGLLPLLLLASFCKASRDVYYNKFGVECEQHQENCAQDRCIFMGWDQLCTQCVPGYVPINGKCQKYP
**Group 4**	6	69	FICSQASNGRCTECNVSTHEMYVFTNPDVSAAEKCILCSDTVGFNGYKGASGCLDCLPPITDKIGIALC
**Group 4**	7	60	CTKDPSKGLCTACPASAKSFLFYGGCYDRDSHIGSRLCSRIGSGVCEQWNSQFDWIFSKN

MEME group	MEME motif ID	MEME motif length (aa)	MEME motif sequence
**Group 5**	1	185	STCQPCQQGCAFCMDGEHQFPYRCLSGGCAQGYYFHPYAQVCMKCPEGCMDCYYDEAHSIPVCERCTDSSQ LTVGPKIYGIWCSDLPPKSCKEDEFQENGFCIPCYLAIMGCIGCSDRFRCTECQPGFYLYDNICVRCDDNCLACD GPGTNCKNCWDGFVLQNGTCVPVGEVIPNCLHADNTTDP
**Group 5**	2	113	MNCPRDHYLLGNMCVECDDAMKWCLECRGPGACTRCKAGYYLDHGYCKPCDHTCERCNGPGKCLECSENHC RNQDGVCDEPGDVYEGCIRCVHDTGKCMQCHEGYFHNGKGFT
**Group 5**	3	110	GFYVADGACKKCVDNCQLCKGPDISNCMLGFEGYRYDSYKNQIEKCQDPNCERCAFTAKYCGTCKLGFYISPDD QKCAKGQVENCMVYVYGQSKCSECIHGYGFSEGGDP
**Group 5**	4	44	KLDLNFSGRGPGVIAAIVILVLIIIGFIVAIPFIVKVLVRRGVR
**Group 5**	5	69	EGIKGEYFDDICEFGVCVQGFYFAYGDSKLLEPPEGASMMKQFKNNVRVGRTRSGQVFAISLVMGYYLH
**Group 5**	6	46	CTKCKDNCYSCKNETECERPASGYYVNPSTGEPAPCTDHNCDICAD
**Group 5**	7	25	RSRLMKIDRGSEDHNEFELEEPDLL
**Group 6**	1	250	MICQDQYKIDVETGISVPVPKGRWYMRIAHRKLWDSPKIHSDPCPKPDRCWACEDGYFLGWYGDKEAKAILTGD YEGQDWKGVDVCLKNTESGKADTPATCTECQEGYYLIPEDPKSEILPDGRIGPHFPNMRCENKKKDYPLEENAN GNRENRAYDDFHPNELGKAVQLQKNSKGYNDEKLKPNKDISEDSAPVYSYEDQILEPPAHKVCKDGYYKESSNS IACYPGFDNRLSDGISANAVIQEGTRQY
**Group 6**	2	192	QQLAIVDDSKFCGDDKAGYQPVDGFMLDNKERVPVNGTPGPNVRSATVGERFEQSKRCQQGYYYPGNRLARS VGNGKDTKSANIQVADEDGYSLPADPDVLNKPDVDVDPQQISNCLPGYYNSRDKCVKCDAGYNWIANEPNCVV AAPPSNGQDSNQSYVEGSGCGRGYGMRGGLSPPAIIGDSVVVIFRVD
**Group 6**	3	69	YAGELPINGQCKQIVRMARRTIFCKKVNGTVLVEDKVIERGLFFYFRRKGGRYHVDNEPGQKLCIQEHE
**Group 6**	4	34	MLLTAFCIMASVLAATCTVEANTRDCAESKCEMV
**Group 6**	5	25	LCFDGYGPNETGSCTPCPDGKGGPN
**Group 6**	6	25	MLMSMFVIMLLVRAATCAVEDVDRD
**TMK-like**	1	250	RVICTRCKDKHIPSEDGSRCIQSCNDSRSESGNCKAGSCTAMGSLYCSRCALDDEWPINGACTGHDTDYVCEK GKGVCTGCLRGYMLYMGGCYDPAAIPGSGICRELLTVGGRTYCKQCNLAGEFPINGLCTKDVPDGVSCSSGLCV ICPENYFFHEGGCYDITTTPGSGICEVLGTGRDAGTCRRCRPGYRHNKETLTCMSCAVSNCASCEIDVKYCTECI QPYTLTGVGAGAYCTKPCKRVGQDACSW
**TMK-like**	2	250	PPDRSSVSDSCVPCTENSDTLKGIPHCKSCKVSNGLLVCVACEQFYRPMNGGTECVQTCVEAEADPKEHRCRK DKCDVLGTFYCSQCAADAEGPVNGVCASVAAGVCIQYKQANGTCKYCTHGYIKYKGGCYEAGSFEAGDICDAQ DVVTSGAGSFCAKCKQKDEVPVDGECKVSDDCTVKGDGICRSCKQGVKSFQITKDGSPATVACFTKCPDGMYE SAEFCQMCDKSCKTCQDSAAKCTGCADGFYL
**TMK-like**	3	250	HCGDGMFLFEGACYTKGRAPGTSICGDTTESTSGCNACEAGFSKTDKGCVRCSDPNCGACVDDPNTCTACRA GYYLQSTSCIACHPACKTCDGMGADKCKECAEGYFGTFATPGLGACLACSDTSGRDSWVGVSGCRLCEAPKVA GPALCKDCLPGYKALSTGTEVTCHAVCPMTYYEAADSCIPCAIDNCATCTSTRCTSCVPEHIWNGEECVPKICPP QCLCREQSIACIGCNENAVEVRPTHSSASR
**TMK-like**	4	250	QDGYYASGNSCVACTPGCKKCDSTGCKVCKDRFALRNNICIEECPVGLLSTSCAAGYCNVNGTLACSRCALLSS VIIDGKCKANDDACIRTTPSSGTCAACKDTHLLYYGGCYAKGAAAGTAICDSQHQATLGPYTICKQCKRDGEVPID GSCVALANTRAIRSQCVAATPSPTSCTRCGPRFFLHAGGCYEIGRSPGTVLCAVAVDSAVAGVCTSCASGYYST GLLDPTKESCILCNDTGATNGYTGVD
**TMK-like**	5	250	RLADRVGPVVCLACESGRKALTLDGMLLCPRDCPTEGFWTSGRECIPCAPGCLRCTSATTCSACSQGYYSTQS SVSAPSDCRPCDDVTSQNGFTGIPHCAVCAADRARAGVTCHACAEGYGLSGGSCSPCTAAGCARCDGGVCTS CKRGWHLAGDRCLACPSPCAACSSAASCLACADGYFQATLLGGPRDCVPCDQTAPQGLQMAGIHGCGLCQAQ AWSGAVLCLRCADGNLPLENYCGGSSVKRRLGP
**TMK-like**	6	221	KVCEEGIGPGKCIYGGCTVRDNTFCSRCSEADEVPIDGVCSLPRERARICLNMSSGRCLRCAATHLLYYGGCYKI TQPVIEICKQDHQIVEKGFAYCKACNDAGEYPVDGSCTAELDGNTCTSSKCTACGSGYFMHRGICMRQEFHSDI CARVGAGVCEECGKSYFKNPVASTAIDSCIHCSDTAGSNGYTGIDHCDTCLPPKAAGPAHCTDCADRYILNE
**TMK-like**	7	250	DKKICTPFDPPNGMCSNCAKGHLLFQGGCYSVAEPLGQEVCAETSEVQERTVCVRCAQPGFVPVSGVCLRYDA QEVAAVGCVTSRGSAVTPTDTTCASCGAKAFLYKGGCYSNETSPGNVICDELDGTGGCKVCRNKYFRSGSTCA PCHQSCETCSSGAADSCLSCKKDVFFEVFSGGRGMCKPCNDPVATITIVGVANCERCSPKPSGTMPICTKCTTG HVLKGNHCVQVCHEGAEGPGQCKTGHCNTI

MEME group	MEME motif ID	MEME motif length (aa)	MEME motif sequence
**VSP-like**	1	250	PAKRRLQYSDGAKGSCVPQCVQDSSCAECGLTVGGTSYCSRCVKSTEYPNNGACTSSSSRAAGIACANVAAGR<br>CVACSSNSFSLNGGCYTSTLLPGKTVCTKASDGWCDSTVAGYGITYNGTLLTCPTNCAMCSSGVCSACNSGFYL<br>EKGACKACSKGCATCPHGEACFDCLAGYYFSESTCKACHKAISKCSLCVVPEGAVKPICLEYDNTSKKSALSSG<br>AISGIAIGVVLVVGGVAAVLVWFFVFRKK
**VSP-like**	2	120	QIESQICQAMQNNVCTTCNSSTKEVFTKNNGCWRCGDSANGGIEGCQRCEMKDSGLQCLECRDLYLSLDKRSC<br>LASCPKGQKGIQGSSSSVHSCACDDGSYLKDGKCVGCAVENCAECDE
**VSP-like**	3	149	IMFFCTECEQDYQPIFPKNVKTVDEKARRAGCVKSPMGGWCTKCNDGYFLFYGGCYQLDQYWKRTICHRAERG<br>ICQECAHGKMMYPPIIFTNPNITSPERCIHDGDIVGFGGYSGVYGQQQKNQHRIPWNIIPDNPQMSEHQMTPYYQ<br>R
**EGF-like**	1	137	DYSGQEATKAPCNGRGRCLLKDPGLAGLSADDYACDCYPIYRGGLCQSCDDANAIAAESSSSDALPTCNARAC<br>QDSDGVVCSGQGTCILDVGLDRESYHYRCKCNDGYTRVGHKCVRTECVATIDGSPVVCGGFGKC
**EGF-like**	2	232	FQNLPGQNGCVHSSCVSSDNKLCSGFGACEKTGDTYGCLCASYYTLVDKDCIPTRCLKDGKVCNGGGTCSGEG<br>SSATCSCSQGWTLHGTLCYPSACVSGGTLCGGNGDCQLSDGGSCSCRSGYETVSGGLCISSQCVQRGTDGTV<br>TICGGNGRCVSENGVKPSCICDEGFSLTSDFVCGVPAPSNKSSAGTTAAIVVVVLLVLVAVAGFLVWWFVIRPRK<br>GGPLRERAPRKP
**EGF-like**	3	127	RRNAGYYCDDRGLATGDLLDTCTPEKSAQIGDKCVPVGCLTSNNQESCKNGGTCVKSDGEYHCRCPDMLIAVH<br>GECTSPACMDEELGKVCSGHGTCKTDDPRNIQCTCEQDYTYIAPGRCILSSLVD
**EGF-like**	4	132	PDPQPPLRMATAQDICGNTGQYTTFGDPSNPFITCVPKDASDGNVSAYNGTFSAKPGCVFISKIDKTRRFFCGFL<br>ENVTENLPTNNLPTCAKPSEKPDLPETCTHCPTDFHLVHFGENNKTCMHKDCHDGQS
**EGF-like**	5	69	DNIRNPDINVHVCSGKGVCAYNQLTGRYGCECEDGLEAFGGYCTHPGCAGKVMHNGELKYVECKVYDGS
**EGF-like**	6	113	PDGYEELDGFCLPKACIFEITTLDNQQMRTMCGGKQFGTCVLNATQQSKSYCKCINRYDIIQITNGKCMKRTCLS<br>DSLPGNPFQYVECSGHGKCKGDHIVGYSCNCDENYQTV
**EGF-like**	7	49	LAEACGTGPACVHGDCAYGDGAHFCQCRRGWAGAACDAPRAGFRRVTTS
